# Supplementary material for: Rapamycin Differentially Impacts Germline Stem Cell Quiescence Across Diverse Genetic Backgrounds of Drosophila Melanogaster
Source: bioRxiv. 2025 Dec 4:2025.12.03.689791. Preprint. [Version 1] doi: 10.64898/2025.12.03.689791 (PMC12822713; doi:10.64898/2025.12.03.689791)

bioRxiv preprint doi: https://doi.org/10.64898/2025.12.03.689791; this version posted December 4, 2025. The copyright holder for this preprint (which was not certified by peer review) is the author/funder, who has granted bioRxiv a license to display the preprint in perpetuity. It is made available under a CC-BY 4.0 International license.

Supplementary Figures

Supplemental Figure 1. (A) Length (mm) of larvae at 72 h post-oviposition, treated with yeast paste dissolved with 5% EtOH (solvent control) or rapamycin (20 μM) diluted in 5% EtOH (see Methods). Red-shaded box-plots correspond to lengths from rapamycin-treated larvae and blue-shaded box-plots correspond to lengths from control-treated larvae (n = 19-62 larva). Batches were repeated if n<15. (B) Sensitivity to rapamycin in larva length represented as a $\log_2$ fold change of mean length (rapamycin/control) over the sensitivity to rapamycin in pupation delay (days, Harrison et al., 2024), where Spearman's correlation (r_s = -0.655, P-value = 0.029).

bioRxiv preprint doi: https://doi.org/10.64898/2025.12.03.689791; this version posted December 4, 2025. The copyright holder for this preprint (which was not certified by peer review) is the author/funder, who has granted bioRxiv a license to display the preprint in perpetuity. It is made available under a CC-BY 4.0 International license.

Supplemental Figure 2. (A) Percentage of GSCs w/ elongated spectrosomes in each DGRP line and w^{1118} at 2 dpi treated with rapamycin (200 μM) or 5% EtOH (solvent control). Red corresponds to data for rapamycin-treated flies, and black corresponds to data for control-treated flies (n=158-212 GSCs). (B) The sensitivity to rapamycin in delay in cell-cycle reentry represented as a $\log_2$ fold change of % GSCs with elongated spectrosomes (rapamycin/control) over the sensitivity to rapamycin in developmental delay (days, Harrison et al., 2024), where Spearman's correlation (r_s = 0.4, P-value = 0.223).

Supplemental Figure 1

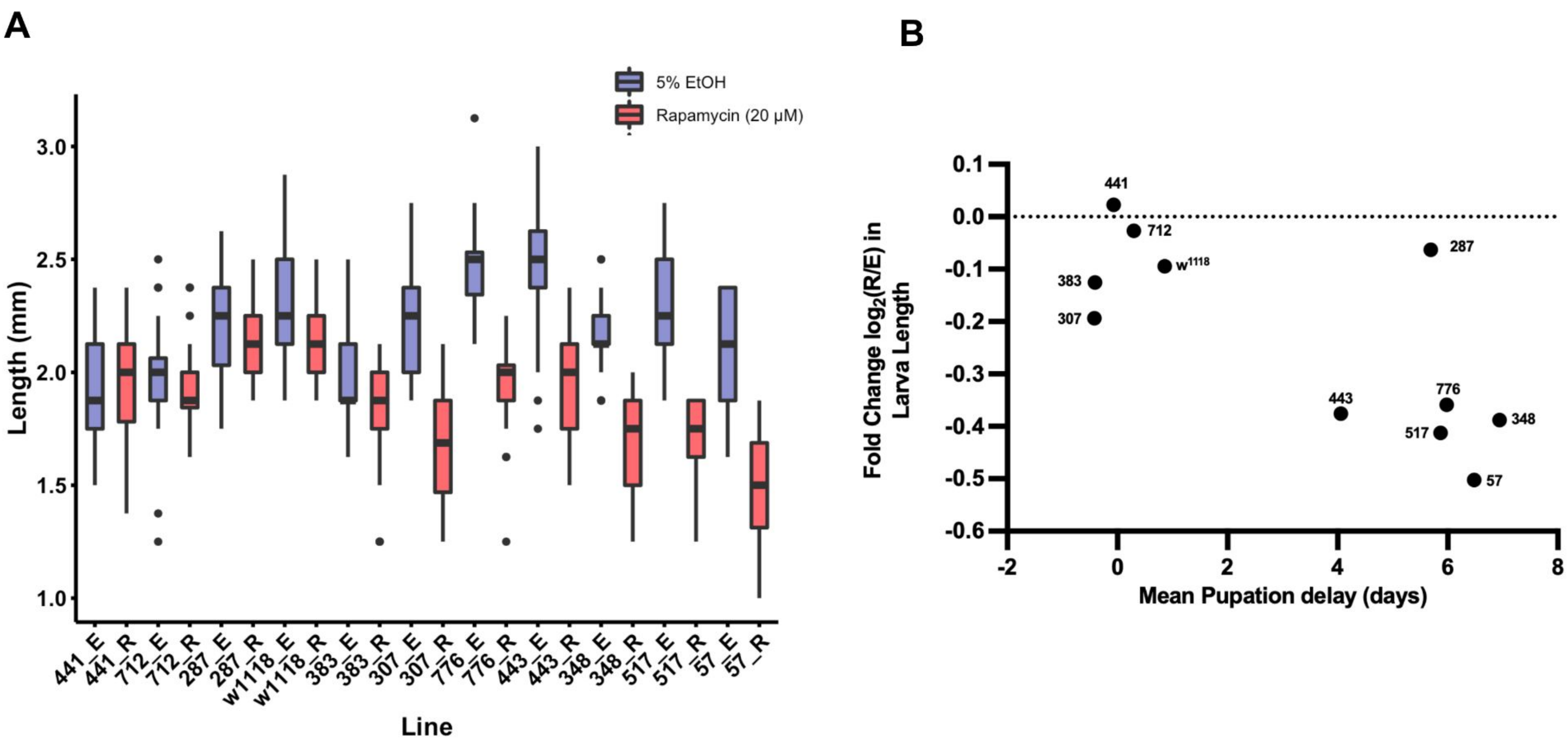

bioRxiv preprint doi: https://doi.org/10.64898/2025.12.03.689791; this version posted December 4, 2025. The copyright holder for this preprint (which was not certified by peer review) is the author/funder, who has granted bioRxiv a license to display the preprint in perpetuity. It is made available under a CC-BY 4.0 International license.

Supplemental Figure 2

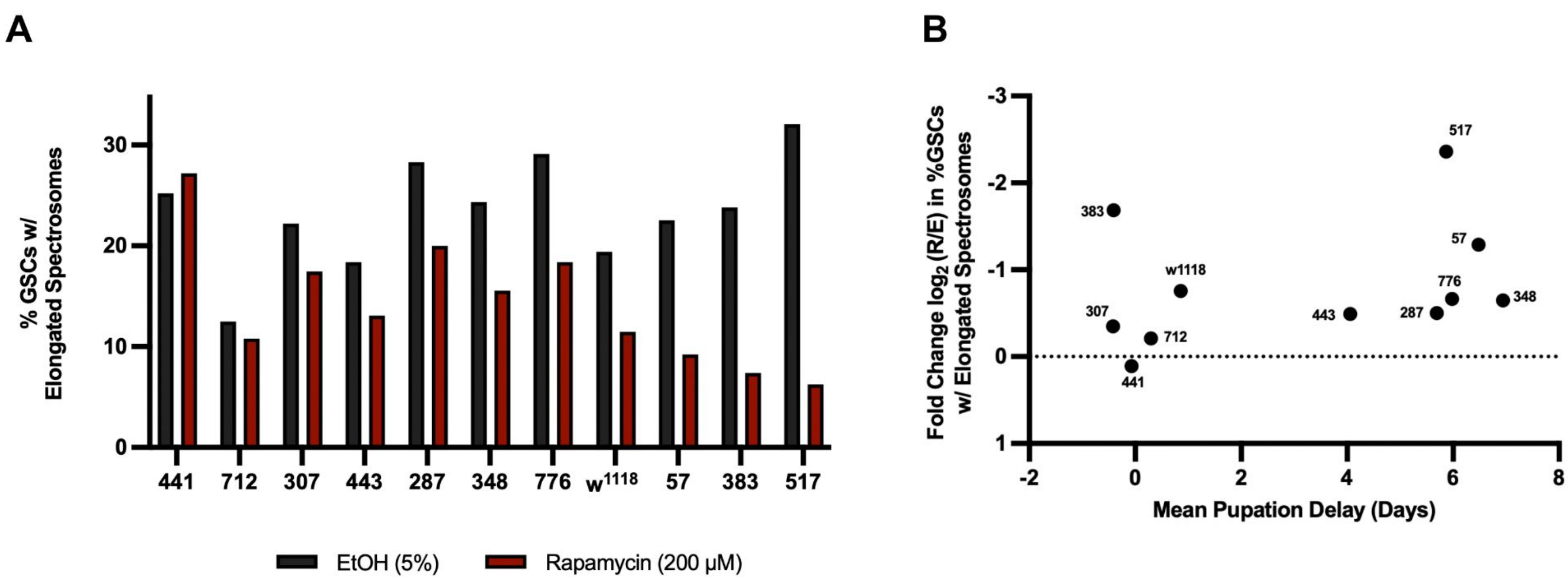

Supplement: Supplementary file 1 [file NIHPP2025.12.03.689791v1-supplement-1.pdf]
